# Genome-wide karyomapping accurately identifies the inheritance of single-gene defects in human preimplantation embryos in vitro

**DOI:** 10.1038/gim.2014.45

**Published:** 2014-05-08

**Authors:** Senthilkumar A. Natesan, Alex J. Bladon, Serdar Coskun, Wafa Qubbaj, Renata Prates, Santiago Munne, Edith Coonen, Joseph C.F.M. Dreesen, Servi J.C. Stevens, Aimee D.C. Paulussen, Sharyn E. Stock-Myer, Leeanda J. Wilton, Souraya Jaroudi, Dagan Wells, Anthony P.C. Brown, Alan H. Handyside

**Affiliations:** 1 Illumina, Cambridge, UK; 2 Department of Pathology and Laboratory Medicine, King Faisal Specialist Hospital and Research Center, Riyadh, Saudi Arabia; 3 Reprogenetics, Livingston, New Jersey, USA; 4 Centre for Reproductive Medicine, Department of Obstetrics and Gynaecology, Maastricht University Medical Centre, Maastricht, The Netherlands; 5 GROW, School for Oncology and Developmental Biology, University of Maastricht, Maastricht, The Netherlands; 6 Department of Clinical Genetics, Maastricht University Medical Center, Maastricht, The Netherlands; 7 Preimplantation Genetics, Melbourne IVF, East Melbourne, Victoria, Australia; 8 Reprogenetics UK, Institute of Reproductive Sciences, Oxford, UK; 9 The Bridge Centre, London, UK

**Keywords:** karyomapping, preimplantation genetic diagnosis, single-gene defect, single-nucleotide polymorphism, whole-genome amplification

## Abstract

**Purpose:**

Our aim was to compare the accuracy of family- or disease-specific targeted haplotyping and direct mutation-detection strategies with the accuracy of genome-wide mapping of the parental origin of each chromosome, or karyomapping, by single-nucleotide polymorphism genotyping of the parents, a close relative of known disease status, and the embryo cell(s) used for preimplantation genetic diagnosis of single-gene defects in a single cell or small numbers of cells biopsied from human embryos following in vitro fertilization.

**Methods:**

Genomic DNA and whole-genome amplification products from embryo samples, which were previously diagnosed by targeted haplotyping, were genotyped for single-nucleotide polymorphisms genome-wide detection and retrospectively analyzed blind by karyomapping.

**Results:**

Single-nucleotide polymorphism genotyping and karyomapping were successful in 213/218 (97.7%) samples from 44 preimplantation genetic diagnosis cycles for 25 single-gene defects with various modes of inheritance distributed widely across the genome. Karyomapping was concordant with targeted haplotyping in 208 (97.7%) samples, and the five nonconcordant samples were all in consanguineous regions with limited or inconsistent haplotyping results.

**Conclusion:**

Genome-wide karyomapping is highly accurate and facilitates analysis of the inheritance of almost any single-gene defect, or any combination of loci, at the single-cell level, greatly expanding the range of conditions for which preimplantation genetic diagnosis can be offered clinically without the need for customized test development.

## Introduction

Preimplantation genetic diagnosis (PGD), following in vitro fertilization (IVF), preimplantation embryo biopsy, and genetic analysis of a single cell or small numbers of cells, is now clinically well established as an alternative to invasive methods of prenatal diagnosis for couples at risk of a range of single-gene defects (SGDs) and chromosome abnormalities.^1,2^ PGD of SGDs, however, remains challenging at the single-cell level. This is because DNA amplification is required for mutation detection and in a low, but significant, proportion of single cells, one of the parental alleles randomly fails to amplify. This can make heterozygous loci appear homozygous. This process is known as allele dropout (ADO) and can result in diagnostic errors. Moreover, the extreme sensitivity of the amplification and detection methods used makes them susceptible to contamination. To avoid these potential causes of diagnostic errors, therefore, the current standard of practice is to combine targeted haplotyping of the gene locus by multiplex amplification of one or more closely linked or intragenic informative polymorphic markers with or without direct mutation detection.^3,4^ Because ADO occurs independently at different loci, the analysis of multiple markers and the mutation itself should allow the detection of any ADO, whereas the detection of unrelated repeat alleles indicates that the sample was contaminated.

Developing family-specific tests requires the investigation of candidate markers, most often short tandem repeats (STRs), within the family, to ascertain which markers are informative (ideally with different numbers of repeats for each of the four parental chromosomes), followed by optimization in single cells. A similar approach is preimplantation genetic haplotyping, in which a generic disease-specific test is developed with a sufficiently large panel of potentially informative markers such that at least two or more markers should be informative in a majority of couples.^5,6^ Nevertheless, both of these approaches are labor intensive, time consuming, and costly to develop and therefore limit testing to specialist laboratories.

As an alternative, a comprehensive linkage-based test has been proposed recently in which single-nucleotide polymorphism (SNP) genotyping of the parents, a close relative of known disease status, and the embryo cell(s), following whole-genome amplification, are used for genome-wide mapping, or karyomapping, of the parental origin of each chromosome in the embryo.^7^ By genotyping the parents at several hundred thousand SNPs throughout the genome on a SNP microarray, a dense set of informative SNP markers are indentified for each of the four parental chromosomes. The phase of the alleles for each informative SNP locus along each chromosome and linkage of the risk alleles with the parental chromosomes are then established by reference to the genotype of the relative of known disease status. The parental origin of each chromosome, or the chromosome segment in recombinant chromosomes, in the embryo cell is then ascertained by comparison with the genotype of the reference. Furthermore, to eliminate errors caused by ADO at the single-cell level, the possible genotype calls at informative SNP loci in the embryo are subcategorized and phasing is based primarily on those that could not have arisen by ADO.

The principal advantage of genome-wide karyomapping relative to targeted approaches is that it is applicable to any familial SGD, or any combination of loci, within the chromosome regions covered by informative SNP loci, without the need for development of patient- or disease-specific tests. Another main advantage is that analysis of SNP markers for each parental chromosome also allows for identification of a range of chromosome abnormalities at high resolution, including trisomies of meiotic origin (in which both haplotypes from one parent can be detected in restricted regions of the chromosome) and monosomies/partial deletions (in which only one parental haplotype is detected).^7^ Chromosome aneuploidy is a major cause of IVF failure, miscarriage, and, rarely, affected live births.^8^ Furthermore, several randomized controlled trials have shown significant improvement in rates of pregnancy and live births following testing for aneuploidy in patients undergoing IVF for infertility.^9,10,11^ The combination of accurate linkage-based diagnosis of SGDs and detection of chromosome aneuploidy may, therefore, improve implantation and rates of healthy live births following transfer of unaffected euploid embryos.

Here, we report the outcome of a multicenter validation study in which parental, reference, and 218 embryo samples from 44 PGD cases were genotyped retrospectively for ~300,000 genome-wide SNP loci in a 24-h protocol compatible with the selection and transfer of unaffected embryos without the need for cryopreservation. Each sample was karyomapped, the disease status of the embryo was analyzed blind, and the results were compared for concordance with the original diagnosis based on targeted haplotyping with direct mutation detection.

## Materials and Methods

### IVF and embryo biopsy

All of the IVF clinics providing samples from PGD cycles used standard IVF protocols for ovarian stimulation, egg collection, and fertilization in vitro by intracytoplasmic sperm microinjection to avoid possible contamination. The morning after the egg collection and fertilization by intracytoplasmic sperm microinjection, each oocyte was examined for the presence of two pronuclei indicating normal fertilization and then either cultured to the 6- to 10-cell stage on day 3 postfertilization for cleavage-stage biopsy and removal of a single blastomere or cultured to the blastocyst stage on day 5 or day 6 for trophectoderm (multiple-cell) biopsy. The embryo samples were then transferred to polymerase chain reaction (PCR) tubes and sent to one of the participating laboratories for targeted haplotyping and mutation detection. After 24–48 h following the diagnosis and transfer or cryopreservation of any unaffected embryos, an additional single-cell biopsy was performed on day 4 or a further trophectoderm biopsy was performed on day 6 on embryos no longer of therapeutic use, with patients' informed consent. In some cases, the embryos had been cryopreserved and were thawed and biopsied for retrospective analysis. Finally, in one center in which whole-genome amplification is used routinely before conventional analysis, aliquots of the archived DNA products were used.

### Whole-genome amplification

With minor variations among the participating laboratories, whole-genome amplification from single-blastomere or trophectoderm samples was performed by multiple displacement amplification (MDA) following cell lysis and neutralization with a short 2-h incubation. Typically, single blastomeres were washed three times in phosphate-buffered saline (Cell Signaling Technologies, Danvers, MA) containing 0.1% polyvinylpyrrolidone (molecular weight 360,000 kD) (Sigma-Aldrich, Zwijndrecht, The Netherlands) and subsequently transferred to a PCR tube containing 2 µl phosphate-buffered saline. The last washing droplet served as a negative embryo control. Blastomere samples were then stored at −20 °C. The sample was brought to an end volume of 4 µl with phosphate-buffered saline, and MDA was performed according to the manufacturer's instructions with a short 2-h incubation (REPLI-g Single Cell Kit; Qiagen, Manchester, UK).

### Targeted haplotyping and mutation detection

Multiplex fluorescent PCR and capillary electrophoresis were used for analysis of STR markers using protocols based on previously published methods. Direct mutation detection was achieved by either minisequencing or standard sequencing methods. Typically, this involved lysing the biopsied cells by incubation at 65 °C for 10 min in lysis buffer (50 mmol/l dithiothreitol (GE Healthcare Bio-Sciences, Uppsala, Sweden) and 200 mmol/l NaOH) before amplification. Multiplex PCR for the polymorphic markers and the mutation was then carried out using a multiplex PCR kit (Qiagen Multiplex PCR kit; Qiagen, Venlo, The Netherlands). The PCR reaction was performed in a final volume of 25–50 μl containing 1× multiplex PCR master mix in 20 mmol/l Tricine (Sigma) and primers for the specific condition (Applied Biosystems, Life Technologies, Warrington, UK). The primer concentrations of the individual protocol in the multiplexed PCRs varied between 0.06 and 1 μmol/l per primer pair, with one of the primers fluorescently labeled. All PCR reactions were performed with an initial activation step of 15-min denaturation at 95 °C. The denaturation–annealing–elongation cycles were conducted as described by the PCR protocol for the specific disorder. The PCR products were diluted 10–20 times, and the labeled amplicons were separated by capillary electrophoresis (ABI Prism 3730XL DNA Analyzer; Applied Biosystems, Life Technologies, Bleiswijk, The Netherlands). Fragment lengths were analyzed using GeneMapper software (Applied Biosystems).

### SNP genotyping

For SNP genotyping, 400-ng samples of parental and reference genomic DNA or 8 μl of MDA product from the embryo samples were processed according to the standard protocol, modified to reduce the initial whole-genome amplification and hybridization steps so that genotyping on a 300K SNP bead array (Human CytoSNP-12; Illumina, San Diego, CA) could be completed in less than 24 h, over a period of 2 days. Bead array data were then imported directly into dedicated software for karyomapping (BlueFuse Multi, Version 4.0; Illumina). With genomic DNA samples, call rates of >98% were generally achieved. With MDA products from single blastomeres and multiple-cell trophectoderm samples, call rates were significantly lower, in the 75–95% range. However, karyomap analysis of these samples was highly consistent, indicating few genotyping errors (excluding ADO). Samples with call rates of <60% (for euploid samples), indicating failure of amplification, had a high incidence of erroneous heterozygous calls, which prevented reliable karyomap analysis. These samples were excluded from further analysis.

### Karyomap analysis

The SNP genotype data from the parents, relative, and embryo samples were used for karyomap analysis as previously described.^7^ Briefly, using a child of known disease status as a reference, for example, (i) the parental SNP genotypes were examined to identify all of the informative SNP loci in which one parent was homozygous (AA or BB) and the other heterozygous (AB), (ii) the allele present on only one of the four parental chromosomes was identified, and (iii) each SNP locus (excluding the Y chromosome) in the embryo was phased relative to the reference based on the presence or absence of this allele in the two samples. Finally, (iv) the inheritance of the unaffected or affected genes was ascertained by examining the parental haplotypes in the region of interest, including the gene locus, in comparison with the reference. The use of a grandparent or other relative of one of the parents as a reference allowed the SNP loci to be phased for that parent only and, consequently, karyomapping of that parent's chromosome in the embryo. In addition, for nonsibling references, only those loci in which the reference is homozygous are displayed. This removes any ambiguities from haplotypes present in the reference that were not inherited by the parents and also reduces the amount of available data by ~50% on average.

BlueFuse Multi (Illumina) displays the detailed karyomaps for each parental chromosome as two rows of informative SNPs (colored dots) mapped to their physical location (pter to qter, from left to right) above and below a continuous haploblock bar, all of which are color coded to indicate the parental haplotype. Haplotypes produced by karyomapping are relative to the reference. Blue and orange represent the paternal and maternal haplotypes, respectively, inherited by the reference. Red and green are the paternal and maternal haplotypes, respectively, that were not inherited by the reference. The two rows of informative SNPs correspond to “key” (above) and “non-key” SNPs (below) (**Table 1**). Key SNPs are defined as sample genotypes at informative loci, the phases in which cannot have been altered by ADO, whereas non-key SNPs are those sample genotypes that are most likely to be accurate but will also include any erroneous phasing results caused by ADO. The haploblocks and the position of crossovers in the central haploblock bar were estimated by Hidden Markov modeling using all of the informative SNP data. These haploblocks are used as a guide for phasing—the diagnosis of an embryo is made considering all available SNP information, the call rate of the embryo, and its estimated level of ADO. Finally, “no calls” in the sample are represented by white dots running along the central axis of the haploblock bar.

### Ethical approval

All of the collaborating centers had institutional review board approval—or its equivalent—and patient consent for anonymized genotyping and analysis of parental, reference, and embryo samples.

## Results

### SNP genotyping

SNP genotyping and karyomapping were successful in 213/218 (97.7%) embryo samples from 44 PGD cycles for 25 different SGDs, with various modes of inheritance and an inherited chromosome microdeletion, distributed widely across the genome (**Supplementary Table S1** online). For the autosomal dominant conditions, a grandparent of known disease status (*n* = 21), or in one case, the brother of the “at-risk” parent was used as a reference. In the remaining cases (*n* = 23, with 16 autosomal recessive, 2 X-linked recessive, 4 X-linked dominant, and 1 inherited microdeletions), a child of known disease status was used. All of the corresponding gene loci had large numbers of potentially informative SNPs represented on the bead array in both the 5′ and the 3′ 2-Mb flanking regions (**Supplementary Table S1** online). Furthermore, with the exception of myotonic dystrophy type 1, all of the genes also had intragenic SNPs represented (range: 2–78), and together with Duchenne muscular dystrophy, there were 320 intragenic SNPs across the dystrophin gene on the X chromosome.

With 10 PGD cycles, 47 samples were derived from multiple-cell trophectoderm samples biopsied at the blastocyst stage, and in one case, five biopsied cleavage-stage embryos were processed directly. In the other 33 cases, 166 single blastomeres were biopsied from cleavage-stage embryos, including 10 embryos in which two single blastomeres were biopsied and analyzed separately. Overall, five samples (2.3%)—including two trophectoderm biopsies and three single-cell biopsies—failed to be karyomapped because the SNP genotyping call rates were too low (defined as <60% call rates in a euploid sample), presumably as a consequence of suboptimal or failed whole-genome amplification.

### Karyomap analysis

**Figure 1:**
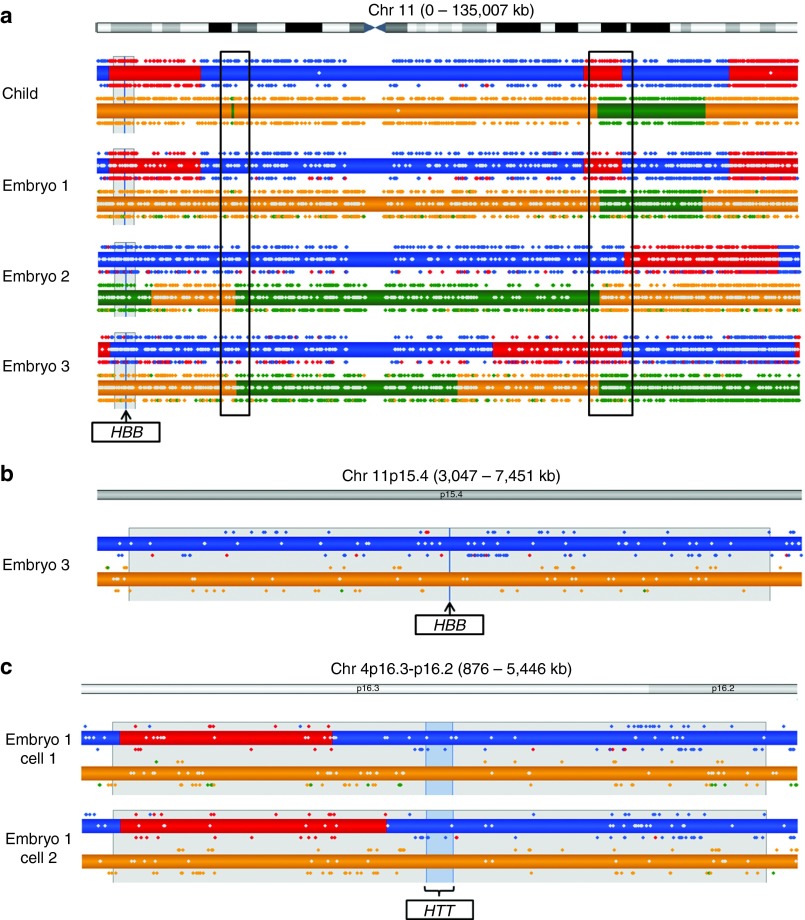
**Karyomaps of single blastomeres biopsied from cleavage-stage embryos.** Paternal haplotypes are represented in blue/red, and maternal haplotypes are represented in orange/green. Haplotypes inherited by the reference are shown in blue/orange. For a detailed description of how the karyomaps are displayed, see the Materials and Methods section. (**a**) Karyomaps for chromosome 11 in three embryos from a preimplantation genetic diagnosis (PGD) case for β-thalassemia and the unaffected child born following transfer of Embryo 1. The sibling used as a reference to phase the SNP calls is a carrier of the affected paternal allele (blue/orange), whereas Embryo 1 and the child born following the transfer of Embryo 1 are unaffected (red/orange), Embryo 2 is affected (blue/green), and Embryo 3 is a carrier of the paternal allele (blue/orange). Note the consistent pattern of key and non-key SNPs (colored dots) above and below the predicted haploblocks. In addition, note the crossovers in both the paternal (upper) and maternal (lower) chromosomes, common to all samples, indicating crossovers in the reference (boxed). (**b**) A detailed view of the β-globin locus (*HBB*) in Embryo 3, including the 2-Mb flanking regions proximal and distal to the gene (gray shading). Note that there are two isolated key SNPs (red dots), presumed miscalls, immediately distal to the gene. (**c**) Detailed karyomap of the huntingtin locus (*HTT*), with 2-Mb flanking regions to the left and right, in two single cells from a cleavage-stage embryo in a PGD case for Huntington disease. Note the recombination of the paternal chromosome (top) in the 2-Mb 3′-flanking region. The location of this recombination upstream of the gene is fixed by the presence of three non-key SNPs (in Cell 1) and by one key SNP and three non-key SNPs (in Cell 2), supporting the presence of the same haplotype as the reference. SNP, single-nucleotide polymorphism.

Typical karyomaps for three embryos, based on SNP genotyping of single blastomeres following cleavage-stage biopsy and whole-genome amplification for PGD of β-thalassemia, are shown in **Figure 1a**. The SNP genotype call rates from the single blastomeres were high, and following karyomapping there was excellent coverage of informative, key, and non-key SNPs across chromosome 11 in each case (except in the pericentromeric regions). In addition, there is general agreement between the key and non-key SNPs, and the haploblock boundaries in successive haploblock regions, with only a few isolated, presumed genotyping errors in the key SNPs and a small proportion of ADOs in the non-key SNPs.

All six chromosomes in the three embryos were recombinant with multiple crossovers between the parental haplotypes. The positions of some of these crossovers, however, appear to be identical in all three embryos. This is an artifact of the initial assumption that the chromosomes in the reference, an existing child in this case, are nonrecombinant. Because this will often not be the case, wherever there is a crossover in the reference chromosome, the karyomapping algorithm will add a “common” crossover to all of the other embryos. By contrast, when a grandparent is used as reference to karyomap across two generations, only crossovers in the embryo are identified (**Supplementary Figure S1** online).

In this PGD cycle, transfer of Embryo 1 resulted in a singleton live birth. With the patients' informed consent, a blood sample was taken from the baby, and genomic DNA was used to confirm the PGD result and for karyomapping. The pattern of crossovers in Embryo 1 is very similar to that of the child, confirming the accuracy of karyomapping at the single-cell level. However, an additional small haploblock was detected on the p arm of the maternal chromosome in the child, which was not present in Embryo 1. This haploblock is generated by crossovers in the child and the reference in almost the same position. The proximal boundary of this haploblock coincides with a common crossover in Embryos 2 and 3 (and others, not shown). Only two key SNPs are present in Embryo 1 in this region, which supports the opposite haplotype for the reference (green, above the bar), and hence this was not resolved by the hidden Markov modeling at the single-cell level.

In each embryo sample, although there is a common crossover distal to the gene locus in the paternal chromosome, the paternal and maternal haplotypes for the β-globin (*HBB*) gene locus are clear. However, in a few samples, presumed miscalled key SNPs in the region of the gene locus need to be taken into account. In the case of Embryo 3, there are two key SNP miscalls immediately 5′ to the gene locus and one non-key SNP (red dots above and below the haploblock bar; **Figure 1b**). In this case, a crude estimate of the probability of a double recombination between the closest flanking key SNPs, consistent with the majority across this region of the chromosome (blue dots), spaced ~415 kb apart is 1.72 × 10^−5^. This is clearly much lower than the chance of random miscalls in this sample examining the haploblock as a whole. The median spacing of SNPs on the bead array is ~6 kb, and, on average, this results in a median spacing of informative SNPs of 26 kb. There is wide variation, as can be seen in these samples, but only a small minority of intervals exceeds 1–2 Mb (data not shown). In general, the position of recombination events between parental chromosomes is identified with high resolution. In three single-blastomere embryo samples (two from one embryo, **Figure 1c**), there was a recombination within the 2-Mb flanking region proximal to the gene in two PGD cases for Huntington disease. The gene locus was phased in all three samples, relying on the presence of informative SNPs proximal to the gene locus.

### Concordance analysis

**Figure 2:**
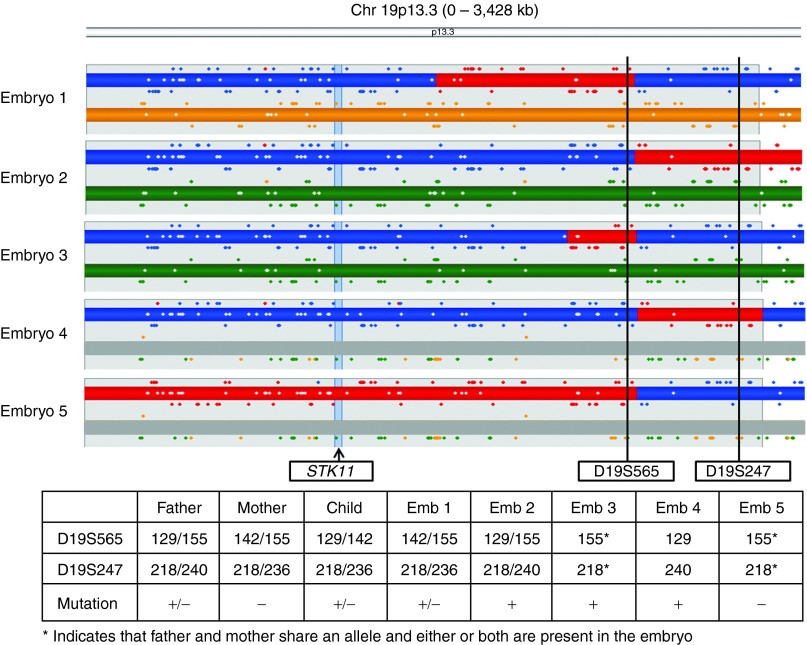
**Detailed karyomaps of chromosome 19, in the terminal p13.3 region, for five embryos from a preimplantation genetic diagnosis case for Peutz–Jeghers syndrome, caused by a mutation in the *STK11* gene.** Also shown is the outcome of conventional testing with two proximal semi-informative STR markers (D19S565 and D19S247) and direct mutation detection. Paternal haplotypes are represented in blue/red, and maternal haplotypes are represented in orange/green. Haplotypes inherited by the reference are shown in blue/orange. The affected child has inherited the mutation in *STK11* from the father. Therefore, the reference haplotype (blue) represents the affected haplotype in this case. Note that there is a common crossover on the paternal chromosome between the two STR markers, which indicates that the affected child used as a reference for linkage had a crossover in this position. Furthermore, there are three additional crossovers in the region on the paternal chromosomes in these five embryos and the maternal chromosome 19 is not present in two embryos (haploblock bar grayed out). This complex pattern of crossovers and aneuploidy detected by karyomapping is completely concordant with the STR alleles (table below) and the presence or absence of the mutation (indicated by the + or − in the STR table). However, Embryos 2, 3, and 4 have identical STR results, and only direct mutation testing identifies Embryo 3 as affected. STR, short tandem repeat.

In 37 PGD cycles, all of the embryos had been unambiguously diagnosed by targeted haplotyping and direct mutation analysis, and blinded karyomap analysis was concordant with the original diagnosis in 156/156 (100%) embryos. In one PGD cycle for Peutz–Jeghers syndrome, however, a recombination between the STR markers was detected along with other presumed ADOs (**Figure 2**). Detailed examination of the STR marker and gene loci in this case showed that it was not possible to find informative STR markers distal to the gene, and the proximal STRs were only semi-informative. The original diagnosis was therefore mainly based on the results of the mutation analysis, which has clearly been affected by ADO in several samples. When the phase of the STR alleles was predicted blindly based on their position, taking into account all four recombinations in the parental chromosomes, there was 100% concordance at the STR level.

**Figure 3:**
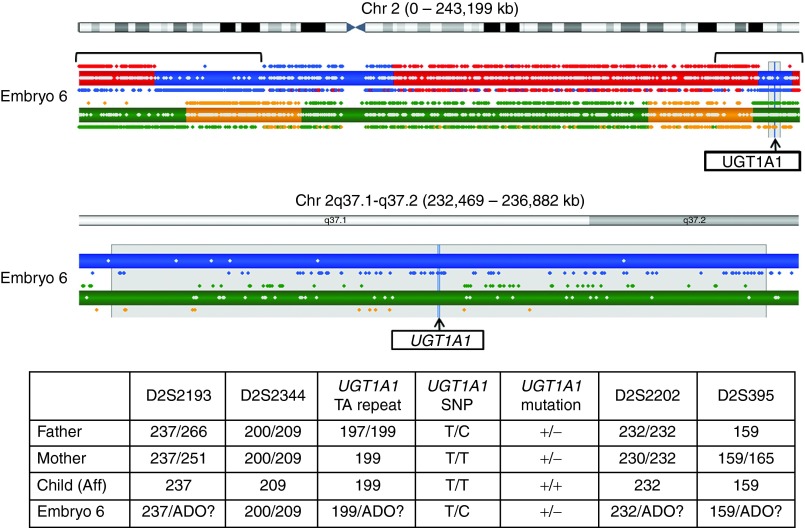
**Comparison of targeted haplotyping and direct mutation detection with karyomapping in a nonconcordant single-blastomere sample (Embryo 6) from a preimplantation genetic diagnosis cycle for Crigler–Najjar syndrome, type 1, caused by a mutation in *UGT1A1*.** Paternal haplotypes are represented in blue/red, and maternal haplotypes are represented in orange/green. Haplotypes inherited by the reference are shown in blue/orange. The father and mother are carriers for this autosomal recessive disorder, and the reference child is affected. This means that both the blue and the orange haplotypes carry the mutation. Embryo 6 has the blue paternal and green maternal haplotypes, indicating a paternal carrier. On the basis of the four flanking STR markers and two intragenic markers, along with the mutation analysis (lower panel), this embryo was diagnosed as an unaffected maternal carrier. In the mutation analysis, + indicates the presence of the mutation, and − indicates its absence. Only the intragenic SNP marker identifies the presence of the normal paternal allele. All other markers are ambiguous and were labeled as suspected ADOs. Phasing of the *UGT1A1* locus by karyomapping is unequivocal (upper panel) and indicates that the embryo is an unaffected paternal carrier. Note that the terminal regions of both chromosome arms have the characteristic karyomapping patterns of key and non-key informative SNPs, indicating regions (marked by square braces) in which both parents have a chromosome with an identical SNP genotype, i.e., identical by descent. ADO, allele dropout; SNP, single-nucleotide polymorphism; STR, short tandem repeat.

In the remaining seven cycles, which were identified by karyomapping as having extensive regions of consanguinity (e.g., **Figure 3** and **Supplementary Figure S2c** online), some or all of the STR markers were only semi-informative, and the results frequently deviated from the expected parental haplotypes. Hence, the original diagnosis was often based mainly on mutation detection. In these cases, karyomapping was concordant in 52/57 (91.2%) embryo samples. However, in five (8.8%) of the single-blastomere samples, the results were nonconcordant. To investigate these discrepancies, the expected STR and mutation site alleles were predicted in a blinded manner, based on the karyomap of each of the embryos. This showed that in two of these nonconcordant samples, there was only a single STR or marker allele that had led to the original diagnosis and that the karyomapping evidence from the many key or non-key SNPs in the region of interest strongly supported the contrary diagnosis (**Figure 3**). Furthermore, because the STR markers were in general only semi-informative, the strength of evidence in the other samples was similarly weak. The reasons for these nonconcordances therefore need further investigation. Overall, 208/213 (97.7%) embryo samples were completely concordant with the original analysis based on targeted haplotyping and mutation detection.

## Discussion

Comparison of SNP genotyping and karyomapping with the current standard of practice for identifying the inheritance of SGDs at the single-cell level by targeted haplotyping and direct mutation detection, in a blinded retrospective analysis of samples from a large series of PGD cases, has demonstrated that karyomapping is both highly accurate and versatile. Frequently, couples become aware that they are at risk of having a child with an inherited disease only following the birth of an affected child. In this situation, the existing child can be used as a reference to phase the informative SNPs and provide linkage to the affected chromosomes in the parents, allowing the embryo samples to be karyomapped. For dominant conditions, however, the existence of an affected grandparent alerts the couple to the risk and it is necessary to use a grandparent as reference. In a broad range of conditions distributed across the genome (**Supplementary Table S1** online) with different modes of inheritance, the use of siblings, grandparents (**Supplementary Figure S1** online), or, in one case, the brother of one of the parents as the reference, karyomapping of single- or multiple-embryo-cell samples was successful and, excluding the consanguineous cases, 100% concordant with the original diagnosis based on targeted haplotyping and direct mutation detection. Thus, the comprehensive methodology of genome-wide karyomapping is applicable to all of these SGDs and greatly expands the range of conditions for which PGD can be offered clinically without the need for customized patient- or disease-specific test development, avoiding delays in patient treatment and reducing costs. Furthermore, for late-onset autosomal dominant conditions, including Huntington disease and cancer predisposition syndromes, karyomapping with a grandparent of known status as a reference allows exclusion testing for the affected grandparental chromosome directly, without revealing the status of the at-risk parent.

Another major advantage of karyomapping, relative to targeted haplotyping analysis, is the ability to detect a range of chromosome abnormalities and their parental origin, including meiotic trisomies, monosomies, and deletions.^7^ Many of the embryos analyzed here showed evidence of one or more aneuploidies, and several partial chromosome deletions were also identified (**Supplementary Figure S2** online). Furthermore, a significant minority of embryos had complex karyotype-wide patterns of aneuploidy consistent with various types of abnormal fertilization. Further work is needed, however, to validate karyomapping for the detection of aneuploidy by comparing results with established techniques, such as array comparative genomic hybridization or next-generation sequencing.

Other applications of genome-wide karyomapping include testing for the inheritance of multiple loci and structural chromosome imbalance.^7^ For example, PGD can be used in conditions such as β-thalassemia to select unaffected, human leukocyte antigen––matched embryos for transfer, so that cord blood stem cells can be collected at birth for transplantation to an existing affected child, and this strategy has been effective in curing these children.^12^ However, at the single-cell level, this requires the development of highly multiplexed amplification protocols with markers distributed across the *human leukocyte antigen* region and at the gene locus.^4^ With karyomapping, all that is necessary is to phase the relevant regions on the short arms of chromosomes 6 and 11 using the existing affected child as a reference. For carriers of balanced structural chromosome rearrangements, particularly reciprocal or Robertsonian translocations, there is a high risk of segregating unbalanced combinations of the normal and derivative chromosomes in meiosis, which can result in infertility, repeated miscarriage, or affected live births depending on the chromosomes involved and the size of the translocated segments. Karyomapping detects both segmental trisomies and deletions in unbalanced embryos (unpublished data). In addition, analysis of at least one unbalanced embryo allows the parental haplotypes proximal to the translocation break points to be determined and normal and balanced embryos to be distinguished, which is not possible by quantitative methods such as array comparative genomic hybridization.

The major limitation of karyomapping for diagnosing inheritance of SGDs is that it is an indirect linkage-based method only and does not include detection of the mutation(s). Thus, karyomapping of embryo samples, without additional testing for the mutation, relies completely on the strength of the evidence for phase within the family. It is not a substitute for careful analysis of the family history and mutation testing of relevant family members. This also means that karyomapping cannot be performed without a reference, which is not always available for every couple. Moreover, karyomapping is not immediately applicable to de novo mutations, most often encountered in autosomal dominant conditions. However, in these cases, it is current practice to establish phase using single sperm cells in affected males, or simply to use linked markers as a backup to direct mutation detection, and the same approaches could be used with karyomapping. Another limitation is that karyomapping cannot detect sequence-identical chromosome duplication that can result from malsegregation of chromosomes during the early cleavage divisions of the embryo. However, meiotic trisomies, which are inherited by fertilization with an aneuploid gamete, can be detected, and these are much more likely to affect pregnancy.^13^

Karyomapping relies on bead array technology and hence incurs higher consumable costs than PCR-based techniques. However, karyomapping allows for significant savings in labor because it does not require the complex, time-consuming workup required for customized tests using highly multiplexed STR analysis. On balance, it is expected that the per-sample cost for karyomapping should be comparable to or less than that for existing methods, depending on the complexity of the analysis.

Recently, targeted next-generation sequencing has been used for mutation detection in single cells as a proof-of-principle study for use in PGD.^14^ In principle, the advantage of whole-genome sequencing for karyomapping would be that analysis of SNPs, and possibly other markers, across the genome to improve diagnostic accuracy could be combined with mutation and copy-number analysis. However, whole-genome amplification from a single cell or small numbers of cells has been shown to introduce a spectrum of different variants at the sequence level, which will be a challenge to filter out and interpret.^15^ Furthermore, such comprehensive preimplantation embryo testing raises various ethical issues.^16^ The advantage of using a microarray platform to genotype a defined set of SNP loci for karyomapping is that there are no incidental findings at the sequence level. Nevertheless, because the SNP density is high, even relatively small partial chromosome deletions may be detected, some of which may have serious clinical consequences. The partial deletions detected in this study were relatively large, and further work will be needed to validate the limits of resolution and clinical significance of these chromosome abnormalities.

## Disclosure

S.A.N., A.J.B., A.P.C.B., and A.H.H. (part time) are employed by Illumina. The other authors declare no conflict of interest.

The karyomapping algorithm is covered by European and Japanese patents (EP1951897B1 and JP5178525B2, respectively; other territories pending) licensed to Illumina.

## Figures and Tables

**Table 1:**
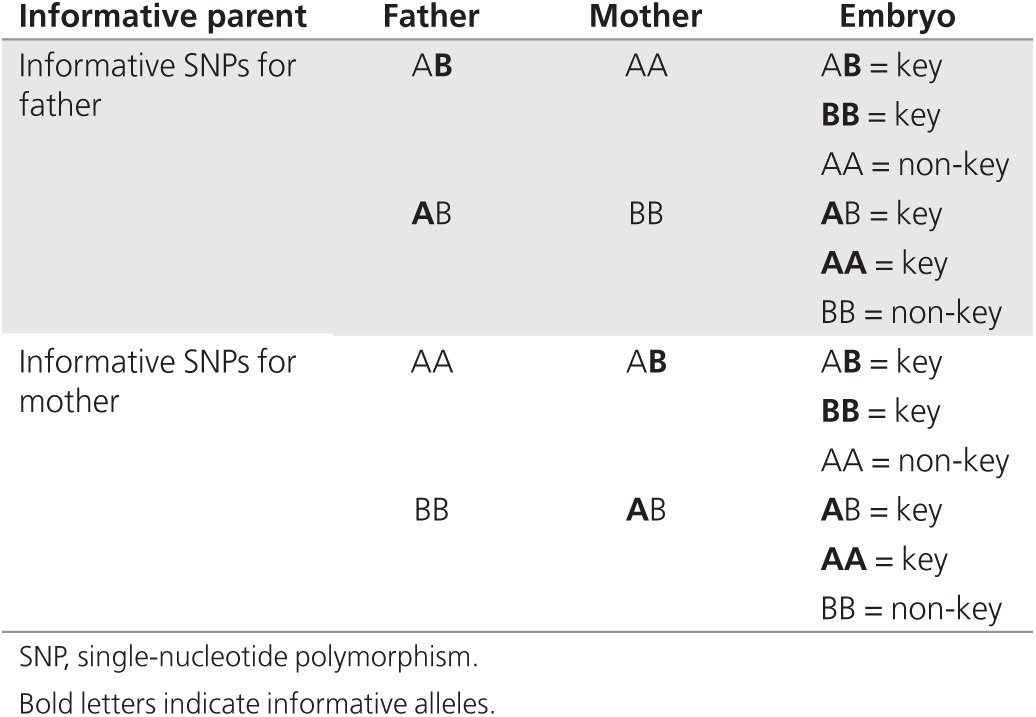
Definition of key and non-key informative SNP loci
