# Supplementary figures and images for: Genome-wide karyomapping accurately identifies the inheritance of single-gene defects in human preimplantation embryos in vitro
Source: Genet Med. 2014 May 8;16(11):838–45. doi: 10.1038/gim.2014.45 (PMC4225458; doi:10.1038/gim.2014.45)

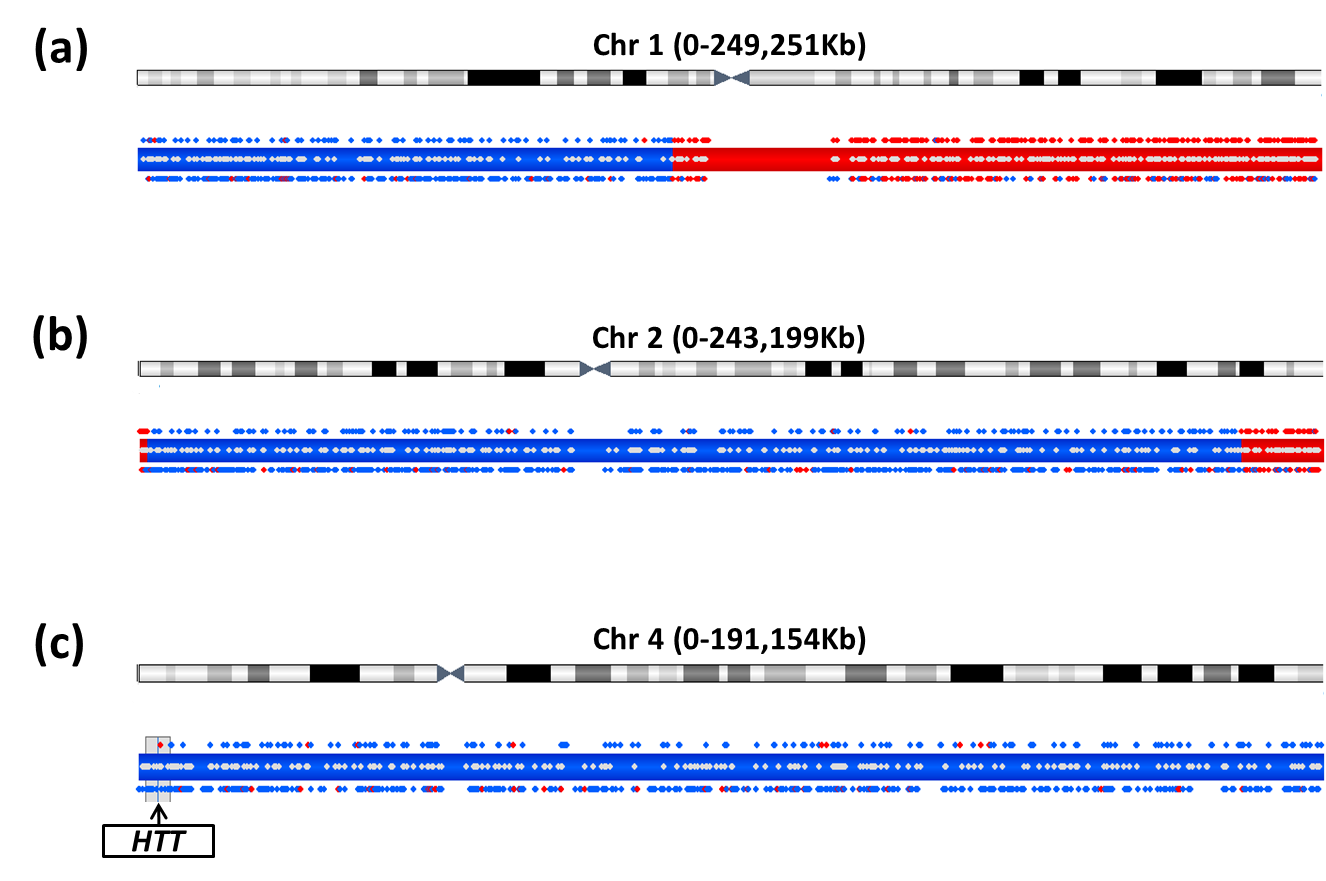

Supplement: Supplementary Figure S1 [file gim201445x1.tiff]

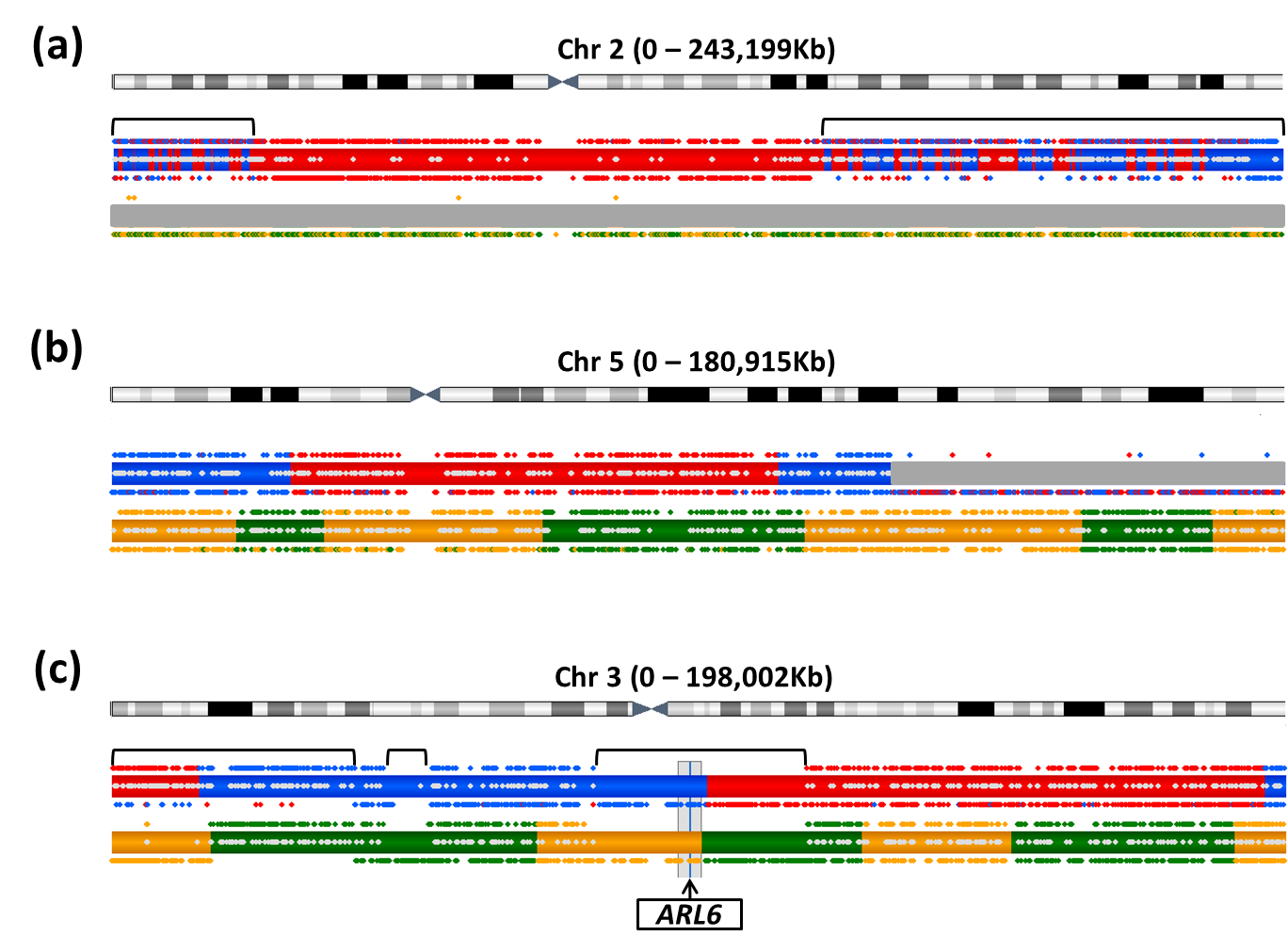

Supplement: Supplementary Figure S2 [file gim201445x2.tiff]
