# Supplementary material for: Genome-wide karyomapping accurately identifies the inheritance of single-gene defects in human preimplantation embryos in vitro
Source: Genet Med. 2014 May 8;16(11):838–45. doi: 10.1038/gim.2014.45 (PMC4225458; doi:10.1038/gim.2014.45)
Supplement: Supplementary Table S1 [file gim201445x3.doc]

**Table S1.** List of PGD conditions with their chromosome locations and SNP coverage in those regions.

| **PGD condition** | **Number of cases** | **Number of Embryos** | **Mode of inheritance** | **Gene/ Locus** | **Phenotype MIM number** | **Chr** | **Region** | **SNP coverage** | | |
| --- | --- | --- | --- | --- | --- | --- | --- | --- | --- | --- |
|  |  |  |  |  |  |  |  | **5'** | **Gene/**  **Locus** | **3'** |
| **Crigler-Najjar syndrome, type I** | 1 | 9 | AR | *UGTIA1* | 218800 | 2 | 234,668,918-234,681,944 | 261 | 2 | 372 |
| **Bardet Biedl syndrome 3** | 1 | 5 | AR | *ARL6* | 209900 | 3 | 97,483,594-97,517,372 | 280 | 6 | 212 |
| **Huntington disease** | 7 | 29 | AD | *HTT* | 143100 | 4 | 3,076,407-3,245,686 | 315 | 14 | 256 |
| **Familial Adenomatous Polyposis 1** | 1 | 4 | AD | *APC* | 175100 | 5 | 112,043,201-112,181,935 | 189 | 20 | 236 |
| **Spinal muscular atrophy-1** | 1 | 6 | AR | *SMN1* | 253300 | 5 | 70,220,767-70,248,838 | 29 | 7 | 98 |
| **Osteopetrosis, infantile malignant 3** | 1 | 9 | AR | *OSTM1* | 259720 | 6 | 108,362,612-108,395,940 | 221 | 7 | 113 |
| **Polycystic Kidney disease** | 1 | 3 | AR | *PKHD1* | 263200 | 6 | 51,480,144-51,952,422 | 274 | 78 | 342 |
| **Cystic fibrosis** | 1 | 3 | AR | *CFTR* | 219700 | 7 | 117,120,016-117,308,718 | 93 | 34 | 55 |
| **Lipodystrophy, congenital generalized, type 1** | 1 | 7 | AR | *AGPAT2* | 608594 | 9 | 139,567,594-139,581,910 | 304 | 2 | 211 |
| **Exostoses, multiple, type 2** | 1 | 1 | AD | *EXT2* | 133701 | 11 | 44,117,098-44,266,979 | 202 | 23 | 346 |
| **Thalassemia, beta-** | 4 | 30 | AR | *HBB* | 613985 | 11 | 5,246,695-5,248,300 | 201 | 14 | 305 |
| **Sickle cell anemia** | 2 | 8 | AR | *HBB* | 603903 | 11 | 5,246,695-5,248,300 | 201 | 14 | 305 |
| **Smith-Lemli-Optiz syndrome** | 1 | 5 | AR | *DHCR7* | 270400 | 11 | 71,145,456-71,159,476 | 213 | 4 | 277 |
| **Breast-ovarian cancer, familial, 2** | 6 | 12 | AD | *BRCA2* | 612555 | 13 | 32,889,616-32,973,808 | 207 | 7 | 151 |
| **Propionic acidemia** | 1 | 6 | AR | *Alpha PCCA* | 606054 | 13 | 100,741,268-101,182,690 | 299 | 46 | 258 |
| **Li-Fraumeni syndrome** | 1 | 1 | AD | *TP53* | 151623 | 17 | 7,571,719-7,590,867 | 250 | 2 | 283 |
| **Breast-ovarian cancer, familial, 1** | 2 | 3 | AD | *BRCA1* | 604370 | 17 | 41,196,311-41,277,499 | 156 | 25 | 340 |
| **Peutz-Jeghers syndrome** | 1 | 9 | AD | *STK11 (LKB1)* | 175200 | 19 | 1,205,797-1,228,433 | 137 | 4 | 307 |
| **Hypercholesterolemia, familial** | 1 | 10 | AD | *LDLR* | 143890 | 19 | 11,200,037-11,244,505 | 281 | 12 | 281 |
| **Myotonic dystrophy 1** | 1 | 6 | AD | *DMPK* | 160900 | 19 | 46,272,974-46,285,814 | 136 | 0 | 108 |
| **Bardet Biedl syndrome 6** | 1 | 15 | AR | *MKKS / BBS6* | 209900 | 20 | 10,385,427-10,414,886 | 324 | 3 | 274 |
| **Duchene Muscular Dystrophy** | 1 | 2 | XR | *DMD* | 310200 | X | 31,137,344-33,357,725 | 226 | 320 | 66 |
| **Fragile X Syndrome** | 3 | 16 | XD | *FMR1* | 300624 | X | 146,993,468-147,032,646 | 279 | 8 | 259 |
| **Myotubular myopathy, X-linked** | 1 | 4 | XR | *MTM1* | 310400 | X | 149,737,046-149,841,615 | 255 | 17 | 356 |
| **Incontinentia pigmenti** | 1 | 4 | XD | *IKBKG* | 308300 | X | 153,770,458 -153,793,260 | 340 | 4 | 246 |
| **Xq deletion** | 1 | 10 |  |  |  | X | 131,336,145-132,612,743 | 156 | 38 | 152 |
|  |  |  |  |  |  |  | **Range** | 29-340 | 0-320 | 55-372 |
